# Effect of obstructive sleep apnea diagnosis on health related quality of life

**DOI:** 10.1186/s12955-015-0253-1

**Published:** 2015-05-29

**Authors:** Serena Iacono Isidoro, Adriana Salvaggio, Anna Lo Bue, Salvatore Romano, Oreste Marrone, Giuseppe Insalaco

**Affiliations:** National Research Council of Italy, Institute of Biomedicine and Molecular Immunology “A. Monroy”, Via Ugo La Malfa, 153, 90146 Palermo, Italy

**Keywords:** Obstructive sleep apnea, Quality of life, Diagnosis, Gender, Hypoxemia, Body mass index

## Abstract

**Background:**

Perceived Health Related Quality of Life (HRQoL) is impaired in obstructive sleep apnea (OSA). To our knowledge, no study has analyzed the effect of OSA diagnosis communication on HRQoL. We evaluated self-perceived HRQoL in patients afferent to our sleep center, in order to examine the effect of the diagnosis disclosure on their HRQoL.

**Methods:**

Two hundred ninety-seven consecutive outpatients (227 M) (mean age 54.1 ± 11.6 yrs, range 23–80 yrs) were evaluated, before first clinical visit and nocturnal diagnostic examination (Time A), and after diagnosis disclosure (Time B), with two self-reported questionnaires for HRQoL assessment: Psychological General Well-Being Index (PGWBI), consisting of anxiety, depressed mood, positive well-being, self-control, general health, vitality subscales, and 12-Item Short-Form Health Survey (SF-12), comprising Physical (PCS) and Mental Component Summaries (MCS).

**Results:**

Comparison of mean HRQoL scores at Time A with reference values, showed worse scores. Mean PGWBI Total and subscales scores improved at Time B. Similar improvement was observed for SF-12 MCS (*p* = 0.0148), but nor for SF-12 PCS. At Time B, Anxiety, Depression and Well-being PGWBI subscales became similar to reference values, while the scores in the other PGWBI subscales and SF-12 remained worse. Comparison between males and females showed higher HRQoL values for males at both times. Score changes were independent from age, gender, BMI, AHI, TSat_90_ and excessive daytime sleepiness.

**Conclusions:**

Diagnosis communication improves patients’ HRQoL, regardless of the severity. Changes in HRQoL after diagnosis disclosure may be due to patients’ motivation for medical check and diagnostic expectations.

## Background

Obstructive sleep apnea (OSA) is characterized by repetitive episodes of complete or partial interruption of ventilation during sleep, which is caused by a collapse in the upper airway. Obstructive apneas are associated with ongoing increasing respiratory efforts, intermittent hypoxia, systemic and pulmonary arterial blood pressure fluctuations and sleep disruption. Approximately 9 % of women and 24 % of men in the general population have sleep-disordered breathing and most of those affected remain undiagnosed [1]; factors that increase susceptibility to the disorder include age, male sex, menopause, obesity, craniofacial abnormalities, family history, and health behaviours such as cigarette smoking and alcohol use [2].

Patients with OSA may exhibit several typical symptoms including habitual snoring (often disruptive for bed partners), feeling of unrefreshed awaking, excessive daytime sleepiness (EDS), psychological disturbances [3, 4], cognitive [5] and emotional disorders [6, 7] that may also give rise to family and social conflict [8].

Perceived Health Related Quality of Life (HRQoL) are deteriorated in sleep-disordered breathing. OSA patients often report a poor HRQoL in social, emotional, and physical domains [9, 10] partially explained by overlapping symptoms including fatigue, loss of interest, decreased libido, and poor concentration [11]. Although many studies have examined the HRQoL in OSA patients, highlighting its deterioration [7, 8, 10, 12, 13], as far as we know, there are no studies that examine the effect of OSA diagnosis on HRQoL. Most studies have assessed patients’ mood and HRQoL retrospectively, after a diagnosis had already been disclosed, therefore missing a comparison with the previous phase in which patients were unaware of the diagnosis [7, 8, 10, 12, 13].

We have already evaluated the HRQoL of patients at the first time visit, pointing out that EDS plays a major role in determining a deterioration in HRQoL [14]. In order to explore whether, and to what extent, disclosure of the OSA diagnosis affects patients’ HRQoL, an assistant psychologist administered unspecific disease questionnaires before the first examination and immediately after diagnosis disclosure.

Aim of this study was to evaluate self-perceived well-being in patients referred to a laboratory for sleep related breathing disorders, and to examine the effect of the diagnosis disclosure on their HRQoL.

## Methods

We performed a study involving patients afferent to our Sleep Laboratory for OSA. Out of 450 consecutive outpatients, 297 completed the study (70 F, 227 M), age 23–80 yrs (54.1 ± 11.6). Patients with a prior diagnosis or treatment for OSA were excluded (n. 84), as were subjects who did not provide consent or did not complete full diagnostic process or questionnaires (n. 66). Patients affected by psychiatric or neurological diseases, diagnosed and pharmacologically treated, were also excluded (n. 3). Subjects underwent a detailed evaluation that included clinical history focused on sleep-related symptoms. The local ethical committee approved the protocol and all subjects gave their written informed consent prior to their inclusion.

For HRQoL evaluation, the Italian versions of Psychological General Well-Being Index (PGWBI) [15], and 12-Item Short-Form Health Survey (SF-12) [16] were administered. These tools were given together to guarantee a further extension of HRQoL measures.

Nocturnal monitoring was performed with a portable computerized system (Somté or Somnea Compumedics Inc.; Abbotsford, VIC, Australia). The recorded signals were airflow, snoring, thoracic and abdominal effort, limb movement, body position, arterial oxygen saturation, pulse rate, and pulse waveform. Duration of recordings was at least 6 h. Apneas and hypopneas were visually scored. According to American Academy of Sleep Medicine Task Force (AASM) standard criteria [17] apneas were defined as lack of airflow for at least 10s and hypopneas were defined as discernible reductions in airflow or thoracic and abdominal movements for at least 10s followed by an arterial oxygen saturation fall >3 %. Apnea-hypopnea index (AHI) was calculated as number of apneas and hypopneas per hour of estimated total sleep time. OSA severity is defined as mild, 5 to 15 events per hour; moderate, 15 to 30 events per hour; severe, greater than 30 events per hour [18].

Percent night-time with O_2_ saturation <90 % (TSat_90_) was evaluated.

### Questionnaires

The Psychological General Well-Being Index (PGWBI) was used to measure perceived psychological well-being [15, 19]. The responses to 22 questions are arranged in six subscales: anxiety (five items), depressed mood (three items), positive well-being (four items), self-control (three items), general health (three items) and vitality (four items). Item responses are rated on a six-point Likert scale ranging from 0 (highest possible distress) to 5 (completely healthy status). The six-scale scores were computed by summing the relevant items. Higher scores indicate better health. The six scales can be further summed to provide a global index score representing one’s comprehensive subjective well-being ranging from 0 to 110. A global score <60 suggests a Severe Distress; from 60 to 72 suggests a Moderate Distress; and >72 suggests No Distress. It was already used to evaluate HRQoL at first time visit in our sleep centre [14].

The 12-Item Short-Form Health Survey (SF-12), is the shorter health self-administered questionnaire derived from the SF-36, allowing faster assessment of patients and producing physical and emotional component summaries without any substantial loss of information compared to the SF-36 [16, 20]. Two subscales are derived from the SF-12: the Physical Component Summary (PCS) and the Mental Component Summary (MCS). The PCS includes questions about physical functioning, role limitations due to physical health problems, bodily pain and general health. The MCS includes questions about vitality (energy/fatigue), social functioning, role limitations due to emotional problems, and mental health (psychological distress and psychological well-being). The PCS and MCS are standardised to a mean of about 50, with a score above 50 representing better than average function and below 50 poorer than average function [20].

The Italian version of Epworth Sleepiness Scale (ESS) [21, 22] was administered to assess daytime sleepiness. Patients rated the likelihood of falling asleep in eight specific situations on a 0–3 scale, with 0 meaning no chance at all of falling asleep, and 3 representing a high chance of falling asleep. Thus, the scale goes from 0 to 24. A score > 10 suggests EDS.

### Study procedure

Patients’ HRQoL was assessed at the first visit (Time A), and immediately after diagnosis disclosure (Time B). The study affected neither the patients’ diagnostic procedure and care routine nor the timing of diagnosis communication. The patients underwent the sleep study 3–18 days after first visit. Between first visit and diagnosis communication 15–30 days occurred. In order to reach uniformity and to avoid an effect on responses, questionnaires were administered in the same order: ESS only at Time A, and PGWBI, followed by SF-12 at Time A and Time B.

Diagnosis of OSA was considered disclosed after the physician had informed the patient about the nature of the condition and therapeutic procedures, when appropriate. Treatments were recommended for OSA patients with an AHI ≥15, regardless of symptoms, and for patients with an AHI ≥5 events per hour accompanied by symptoms (EDS, impaired cognition, etc.) or comorbidities.

After second administration (Time B), the patients were briefed about HRQoL questionnaires results evaluated at Time A.

### Statistical analysis

Difference between means was assessed by the non-parametric Wilcoxon test. Difference between means at Time A and Time B was evaluated by paired t-test. Pearson’s chi-square was applied to assess differences in distress categories at Time A and Time B if any. Relationships between selected variables were identified through simple linear regression. Data were reported as mean ± SD. A *p* < 0.05 was considered significant. Statistical analysis was performed by commercial software (JMP 8.0 SAS Institute Inc.).

## Results

Sample characteristics and nocturnal polygraphic results for total population and for males and females are reported in Table 1.

**Table 1:** Summary of patient characteristics and nocturnal polygraphic results

	Total	Female	Male
**Subjects, No.**	297	70	227
**Age, yrs**	54.1 ± 11.6	58.9 ± 10.2	52.6 ± 11.7*
	(23–80)	(36–80)	(23–80)
**BMI, kg/m** ^**2**^	31.3 ± 6.2	32.8 ± 7.1	30.8 ± 5.9
	(19.1–50.8)	(19.1–50.8)	(19.5–49.9)
**AHI, n/h**	35.5 ± 25.6	34.1 ± 29.3	35.9 ± 24.4
	(0–118)	(0–118)	(0–97)
**Normal, No. (%)**	25 (8.4)	10 (14.3)	15 (6.6)
**Mild, No. (%)**	63 (21.2)	17 (24.3)	46 (20.3)
**Moderate, No. (%)**	61 (20.5)	12 (17.1)	49 (21.6)
**Severe, No. (%)**	148 (49.8)	31 (44.3)	117 (51.5)
**TSat**_**90,**_ **%**	16.6 ± 21.3	19.4 ± 25.2	15.8 ± 19.9
	(0–90)	(0–81.8)	(0–90)
**ESS score**	9.5 ± 5.2	9.3 ± 5.6	9.5 ± 5.1
	(0–24)	(0–22)	(0–24)

Data are presented as mean ± SD (range). BMI, body mass index; AHI, Apnea hypopnea index; TSat_90_, percent night-time at less than 90 % oxygen saturation; ESS, Epworth Sleepiness Scale.

*p < 0.0001 vs Female.

Table 2 shows PGWBI and SF-12 reference values as reported by the questionnaires manuals [15, 16], and sample scores at Time A and B. Mean HRQoL scores at Time A were worse than reference values. At Time B Anxiety, Depression and Well-being PGWBI subscales improved and became similar to reference values, while the scores in other PGWBI subscales and SF-12 remained worse.

**Table 2:** Health-related quality of life data by PGWBI and SF-12

	RV	Time A	Time B	*p* values Time A vs RV	*p* values TimeB vs RV	*p* values Time A vs Time B
**PGWBI**	78.0 ± 17.9	70.4 ± 17.3	74.4 ± 17.9	*p* < 0.0001	*p* = 0.0004	*p* < 0.0001
**Anxiety**	17.3 ± 5.0	15.8 ± 4.7	17.1 ± 4.6	*p* < 0.0001	NS	*p* < 0.0001
**Depression**	12.4 ± 2.6	12.0 ± 2.7	12.3 ± 2.6	*p* = 0.0053	NS	*p* = 0.0009
**Well-being**	11.8 ± 4.0	11.2 ± 3.8	11.8 ± 3.8	*p* = 0.0113	NS	*p* < 0.0001
**Self-control**	11.8 ± 2.7	10.9 ± 3.0	11.3 ± 3.1	*p* < 0.0001	*p* = 0.0016	*p* = 0.0104
**Health**	11.1 ± 3.0	9.5 ± 2.8	10.2 ± 2.7	*p* < 0.0001	*p* < 0.0001	*p* < 0.0001
**Vitality**	13.4 ± 4.0	10.9 ± 4.0	11.6 ± 4.0	*p* < 0.0001	*p* < 0.0001	*p* < 0.0001
**SF-12 PCS**	50.0 ± 9.5	45.0 ± 9.7	45.5 ± 9.9	*p* < 0.0001	*p* < 0.0001	NS
**SF-12 MCS**	50.1 ± 10.0	45.4 ± 10.8	46.5 ± 10.8	*p* < 0.0001	*p* < 0.0001	*p* = 0.0148

Data are presented as mean ± SD. PGWBI, Psychological General Well-Being Index; SF-12, 12-Item Short-Form Health Survey; PCS, Physical Component Summary; MCS, Mental Component Summary; RV, Reference Values; Time A, first time visit; Time B, after diagnosis communication.

PGWBI scores at Time B were improved compared to Time A, similar improvement was observed for SF-12 MCS (*p* = 0.0148), but not for SF-12 PCS (Table 2). Applying criteria for minimal important change (4 points difference for PGWBI and 2 points for each summary of SF-12) [16, 23] the difference observed at Time B for mean PGWBI Total score was clinically meaningful. Moreover, comparing Time A with reference values, the mean PGWBI Total Score was clinically lower. Conversely, at Time B, the mean score was not different from reference values. No clinically meaningful difference was observed in SF-12.

Table 3 shows sample distribution for distress categories at Time A and B, as reported in PGWBI Manual [15]. Distribution among categories was different between Time A and B (chi-square = 6.99; *p* < 0.04). At Time B subjects with No-Distress increased, while those with Severe and Moderate Distress decreased. There were no changes in distress categories in 136 subjects with No-Distress, 29 with Moderate Distress and 52 with Severe Distress.

**Table 3:** Sample distribution for PGWBI descriptive distress categories before and after diagnosis

	No-distress	Moderate distress	Severe distress
**Time A**	145	73	79
**Time B**	177	56	64

Contingency table reporting number of subjects for distress categories at Time A and Time B.

Comparison between males and females showed better HRQoL values for males at both times. After diagnosis communication males improved all scores except one (SF-12 PCS), while females improved only in Total Score, Anxiety, Well-Being, Health and Vitality PGWBI subscales (Table 4).

**Table 4:** Gender effect on health-related quality of life at Time A and Time B

	Time A		Time B		Time A vs Time B	Time A vs Time B
	Female	Male	Female	Male	Female	Male
**PGWBI**	61.4 ± 18.4	73.2 ± 16.0*	66.7 ± 19.5	76.6 ± 16.8*	0.0002	<0.0001
**Anxiety**	14.1 ± 5.2	16.3 ± 4.5†	15.8 ± 5.0	17.5 ± 4.5†	0.0012	<0.0001
**Depression**	10.8 ± 3.0	12.3 ± 2.5*	11.2 ± 3.1	12.7 ± 2.4*	NS	0.0021
**Well-being**	9.1 ± 3.9	11.8 ± 3.5*	10.0 ± 4.1	12.3 ± 3.5*	0.0197	0.0011
**Self-control**	9.6 ± 3.3	11.4 ± 2.8*	10.1 ± 3.3	11.7 ± 2.9*	NS	0.0355
**Health**	8.4 ± 2.8	9.9 ± 2.7*	9.3 ± 3.0	10.5 ± 2.6†	0.0007	<0.0001
**Vitality**	9.3 ± 4.3	11.4 ± 3.7*	10.3 ± 4.3	12.0 ± 3.8†	0.0047	0.0014
**SF-12 PCS**	39.7 ± 10.2	46.6 ± 9.0*	41.1 ± 9.4	46.9 ± 9.6*	NS	NS
**SF-12 MCS**	40.8 ± 11.4	46.8 ± 10.2*	41.4 ± 11.6	48.1 ± 10.0*	NS	0.0131

Data are presented as mean ± SD. PGWBI, Psychological General Well-Being Index; SF-12, 12-Item Short-Form Health Survey; PCS, Physical Component Summary; MCS, Mental Component Summary; Time A, first time visit; Time B, after diagnosis communication.

*p < 0.0001, †p < 0.01 vs Female.

Linear regression analysis between HRQoL scores at Time B minus Time A, and age, gender, body mass index (BMI), AHI, Tsat_90_ and ESS did not show any significant relationship.

## Discussion

The results of this study show that communication of OSA diagnosis has an impact on patients’ HRQoL. At first visit (Time A), our sample had worse scores than reference values [15, 16]. The assessment of patients, showed a significant improvement in HRQoL as evaluated by PGWBI subscales and in SF-12 MCS after the diagnosis communication, irrespective of OSA severity. At Time B, the scores of Anxiety, Depression and Well-being PGWBI subscales improved and were similar to those of the reference values [15, 16]. Sample distribution for PGWBI descriptive distress categories at Time B showed an increase of subjects in the No-Distress category. Females reported lower scores than males in both questionnaires, and lower improvements at Time B. Using the criteria for minimal important change [16, 23], scores variation was independent of age, BMI, AHI, TSat_90_ and EDS.

As far as we know, no study has investigated the effect of being informed of OSA diagnosis on HRQoL. Most available studies concern the effect of cancer diagnosis communication, but findings are controversial. Some data support a positive effect of the knowledge of a cancer diagnosis [24] whereas others suggest that diagnosis worsens patients’ HRQoL [25]. Differences in the questionnaires, study design and cultural backgrounds could account for these divergent results. A study on effect of multiple sclerosis diagnosis shows that its disclosure improves patients’ psychological well-being [26]. As concerns OSA, Głebocka et al. [27], did not find any significant psychological disturbances in OSA patients, interviewed before starting treatment, as compared to normal healthy adults. The authors attributed this unexpected lack of psychological differences to the rapid mood improvement in OSA patients on anticipation of being diagnosed and taken care of in the hospital setting [27].

HRQoL among subjects afferent to our sleep center for suspicion of OSA was impaired as we already observed in a previous study [14], and improved after diagnosis communication. Splitting the sample by gender, women showed a worse HRQoL than men. Similar results were observed in many other studies, despite they adopted other HRQoL or mood evaluation instruments [28, 29]. Women in a healthy population, report poorer well-being and have a higher symptom complaint rate [30]. Gender differences could be explained by women characteristics such as greater bodily attention, as well as social acceptance for women to express distress [31]. In our sample, after diagnosis communication, both genders showed an improved HRQoL. Male subjects reported improvement in all PGWBI subscales and SF-12 MCS, while females improved PGWBI Total score, and Anxiety, Well-Being, Health and Vitality subscales. Several reasons may be involved in this positive effect. Approach of the clinical staff may be important when patients are made aware of their physical conditions.

Changes in HRQoL scores at Time B were independent from age, BMI, severity of disease (AHI), nocturnal hypoxia and EDS. Dutt et al. [32] observed that the HRQoL impairment was not proportional to OSA severity. D’Ambrosio et al. [10] noted that all dimensions of the HRQoL were significantly impaired in OSA patients as compared to the normal population, but did not observe any correlation between increase in severity illness and HRQoL burden [29]. In another study [33], the authors conclude that the altered HRQoL of patients with sleep-related breathing disorders is a multifactorial phenomenon depending on the interaction among sleep stages, daytime sleepiness and obesity, with no significant contribution of sleep fragmentation, hypoxemia and apnea recurrence. The physiological parameters alone may not be the only determinants of the extent of patients’ disturbance and they can not be taken as a marker of HRQoL [32].

Since the relationship between symptoms and HRQoL is neither simple nor direct, there is a need for a complete evaluation, considering physiological, emotional, and social impairment of each patient, assessing well-being independently of OSA severity. To examine the effect of the diagnosis communication on HRQoL in patients referred to a sleep laboratory, we have also assessed HRQoL of non-OSA subjects reporting a wide range of symptoms and disease severity that led them to consult a sleep center. Therefore, someone with an experience of poor health and low expectations about his health status might not consider OSA diagnosis as having an adverse impact, but could even feel less distressed after diagnosis, as solutions to perceived symptoms may be found. Conversely, Garr et al. pointed out that someone who generally has good health might experience a significant negative impact on HRQoL from a relatively minor illness [34]. Recognition of OSA implies, in some cases, the use of chronic therapies, like continuous positive airway pressure (CPAP), that could cause significant distress to the patient. However, some implications could be relevant even in case of negative diagnosis, since waiting for an exhaustive diagnosis may keep the patient in a state of psychological distress, especially when further checks are needed to explain the symptoms. So, changes in HRQoL after the communication of OSA diagnosis may depend on health expectations, symptoms perceived, environmental and individual characteristics, patients’ motivation for medical check and the relationship between physician and patient. Nevertheless, the findings suggest that OSA diagnosis knowledge together with disease explanation may promote the process of adaptation to the disease with an improvement in psychological well-being.

Our study has some limitations, the most important being related to the different size of males and females subgroups. Further studies are needed to assess gender differences and to investigate diagnostic expectations at first visit. Despite these limitations, this study provides initial evidence that OSA diagnosis communication has an impact on HRQoL, probably influenced by personal and environmental factors.

## Conclusions

Disclosure of OSA diagnosis affects patients’ well-being. In our sample, diagnosis communication improves patients’ HRQoL, regardless of OSA severity and anthropometric characteristics. HRQoL investigation appears useful for a complete assessment of each patient disturbance, analyzing physiological and emotional impairments. Patients’ motivation for medical check and diagnostic expectations should be taken into account during HRQoL assessment, to schedule specific interventions to support and promote well-being, and to improve patient-physician relationship.

### Ethics, consent and permission

The research has been performed in accordance with the Declaration of Helsinki and has been approved by the Institutional Ethical Committee Palermo 1 dell’A.U.O.P. (Azienda Ospedaliera Universitaria Policlinico “U. Giaccone”).

Informed consent was obtained from all participants to the study.

## Abbreviations

AHI: Apnea hypopnea index
BMI: Body mass index
EDS: Excessive Daytime Sleepiness
ESS: Epworth Sleepiness Scale
HRQoL: Health Related Quality of Life
MCS: SF-12 Mental Component Summary
OSA: Obstructive sleep apnea
PCS: SF- 12 Physical Component Summary
PGWBI: Psychological General Well-Being Index
SF-12: 12-Item Short-Form Health Survey
TSat_90_: Percent night-time at less than 90 % oxygen saturation
